# The Effect of *Lactiplantibacillus plantarum* SHY130 and Konjac Glucomannan on the Physicochemical, Antioxidant, and Sensory Properties of Stirred Yogurt

**DOI:** 10.3390/foods13152324

**Published:** 2024-07-24

**Authors:** Penglin He, Yufei Wang, Jing Yang, Huayi Suo, Jiajia Song

**Affiliations:** 1 College of Food Science, Southwest University, Chongqing 400715, China; 2 Chongqing Key Laboratory of Speciality Food Co-Built by Sichuan and Chongqing, Chongqing 400715, China; 3 College of Food Science and Engineering, Chongqing Technology and Business University, Chongqing 400067, China

**Keywords:** yogurt, synbiotics, konjac glucomannan, lactic acid bacteria

## Abstract

The impact of konjac glucomannan (KGM)-based synbiotics on yogurt quality is not well understood. This study investigated the effects of a synbiotic mixture of KGM and the selected probiotic *Lactiplantibacillus plantarum* SHY130 on the physicochemical, antioxidant, and sensory properties of yogurt. The results showed that KGM significantly promoted the growth of *Lactiplantibacillus plantarum* SHY130. The synbiotics dramatically enhanced the count of lactic acid bacteria in yogurt during the 14 days of storage. Texture analysis indicated that the synbiotic supplement had no impact on springiness and cohesiveness but resulted in notable reductions in hardness, gumminess, and chewiness. The synbiotics did not significantly affect the water-holding capacity and syneresis. While the synbiotics initially decreased yogurt viscosity, it increased with storage time. Furthermore, the synbiotics significantly improved the yogurt’s antioxidant capacity. Additionally, the supplementation of the synbiotics did not adversely affect sensory properties, although the synbiotics containing 0.02% KGM negatively impacted overall acceptability. Overall, these findings elucidate the effects of KGM-based synbiotics on yogurt quality, providing a foundation for developing novel synbiotic yogurt products.

## 1. Introduction

Yogurt is a dairy product produced through the fermentation of milk by lactic acid bacteria (LAB). It is highly valued for its unique taste and nutritional benefits. In recent years, yogurt has gained significant attention as a carrier of probiotics. Probiotic yogurt is supplemented with strains such as *Bifidobacterium* and *Lactobacillus acidophilus*, which offer numerous health benefits when maintained in adequate numbers [1]. Yogurt supplemented with probiotics has been found to exhibit a better texture compared to yogurt without probiotics [2]. However, probiotics are sensitive to various factors, including pH, storage temperature, and processing conditions, which can significantly impact their viability in yogurt [3].

Prebiotics are defined as indigestible substrates that reach the large intestine without being digested by the host and exert a beneficial effect by selectively stimulating the growth or activity of beneficial bacteria in the colon [4]. Among the many ways to protect probiotics, prebiotics can effectively enhance their viability during storage and while passing through the gastrointestinal tract [5]. Konjac glucomannan (KGM) is a water-soluble, non-ionic macromolecular polysaccharide with unique properties [6]. It is extracted from the roots and tubers of Amorphophallus konjac and consists of β-1,4-linked D-mannosyl and D-glucosyl groups in a molar ratio of 1.6:1.0. Certain branch points are found at the C-3 position of the glucosyl and D-mannosyl residues, and some are found at the C-6 position of the glucosyl unit [7,8]. KGM can promote the multiplication of certain microorganisms and prevent antibiotic-induced gut microbiome perturbation in mice [9]. Additionally, KGM cannot be hydrolyzed by digestive enzymes in the gastrointestinal tract, and KGM-based delivery systems protect probiotics from gastric acid, low pH, and intestinal bile salt damage [10]. Therefore, KGM has great potential as a new prebiotic.

Although studies have demonstrated the potential of KGM as a prebiotic, there are currently few investigations on combining it with probiotics to form a synbiotic and incorporating it into yogurt. The purpose of this study is to investigate the effect of KGM-based synbiotics on the physicochemical, antioxidant, and sensory properties of stirred yogurt. First, probiotics with the ability to utilize KGM were screened. Subsequently, the physicochemical and antioxidant properties of KGM-based synbiotic yogurt were evaluated by determining viable bacterial count, pH, titration acidity, water-holding capacity, syneresis, texture, viscosity, color, and antioxidant activity. Finally, this study also explored the effect of KGM-based synbiotics on the sensory properties of yogurt. This study, for the first time, reveals the impact of KGM-based synbiotics on yogurt quality characteristics and provides a scientific basis for developing new synbiotic yogurts.

## 2. Materials and Methods

### 2.1. Materials

The strains (*Lactiplantibacillus plantarum* 17001, *Lactiplantibacillus plantarum* SHY130, *Limosilactobacillus fermentum* H131, *Lacticaseibacillus rhamnosus* 127, *Limosilactobacillus fermentum* 02001, *Lacticaseibacillus casei* 01001, *Lactiplantibacillus plantarum* S58, and *Lacticaseibacillus rhamnosus* T53) used in this experiment were screened from traditional fermented foods (yogurt, cheese, and pickles) in our laboratory, as described previously [11]. The pasteurized milk was provided by Chongqing Guangda Dairy Co., Ltd., Chongqing, China. The starter cultures (*Streptococcus salivarius* subsp. *thermophilus* and *Lactobacillus delbrueckii* subsp. *bulgaricus*) were purchased from DuPont (Wilmington, DE, USA).

### 2.2. Screening of Strains That Efficiently Metabolize KGM

The activated strains with an inoculum volume of 2% (*v*/*v*) were inoculated into an MRS liquid medium containing 0.5% (*w*/*v*) KGM as the sole carbon source. The culture was mixed thoroughly and incubated at a constant temperature of 37 °C. The optical density of the bacterial suspension was measured at 600 nm at 0, 3, 6, 9, 12, 24, and 48 h post-inoculation. Subsequently, the bacterial suspension was centrifuged at 1700× *g* for 10 min, and the supernatant was collected to determine the pH. A medium devoid of a carbon source served as the negative control, while a medium with glucose as the carbon source was used as the positive control.

### 2.3. Preparation of Stirred Yogurt

The yogurt was prepared as previously described [12]. First, 8% (*w*/*v*) sucrose was added to the pasteurized milk and mixed. After it was fully dissolved, it was placed in a 95 °C water bath for 5 min. Once the milk had cooled to 42 °C, 0.03% (*w*/*v*) of a starter culture was added for the control group and experimental group. For the experimental group, 2% (*v*/*v*) of *Lactiplantibacillus plantarum* SHY130 suspension was supplemented, followed by the addition of 0%, 0.01%, and 0.02% KGM. The inoculated milk was then placed in an incubator set at 42 °C for cultivation. After 8 h, the yogurt was removed from the incubator and gently stirred until no visible particles were observed. It was then placed in a refrigerator at 4 °C for a post-ripening period of 12 h. The properties of the stirred yogurt samples were assessed at the storage intervals of 1, 7, and 14 days [13].

### 2.4. Determination of Viable Bacterial Count, pH, and Titration Acidity of Yogurt

To quantify the LAB count in each yogurt sample after 14 days of storage at 4 °C, the method described previously was used [12]. A stock solution was prepared by mixing 1 mL of the sample with 9 mL of 0.09% (*w*/*v*) sterile NaCl solution. After the gradient dilution of the yogurt, 10 μL of each dilution concentration was applied to the MRS agar medium, cultured at 37 °C for 48 h, and then counted. The pH and titration acidity of the yogurt were analyzed using a previously described method [14] with minor modifications. The pH was measured using a pH meter (PHS-320, Chengdu Century Ark Technology Co., Ltd., Chengdu, Sichuan Province, China). For the determination of titration acidity, 10 g of the yogurt was diluted in 20 g of distilled water, and the mixture was titrated with 0.1 M NaOH until a permanent pink color was observed upon the addition of phenolphthalein as an indicator. The results were calculated using the following formula:

$$X=\frac{c\times v\times 100}{m\times 0.1}$$

where X is the sample’s acidity; *c* is the molar concentration of the standard solution of sodium hydroxide (mol/L); *v* is the volume of the standard solution of sodium hydroxide consumed by titration; 100 refers to a 100 g sample; *m* is the mass of the sample.

### 2.5. Determination of Water-Holding Capacity (WHC) and Syneresis of Yogurt

The WHC and syneresis of each sample were determined using a reported method [12]. Briefly, 20 g of the yogurt was transferred into a centrifuge tube and then centrifuged at 4 °C and 3000 rpm for 10 min. Following centrifugation, the supernatant was carefully decanted, and the weight of the separated whey was recorded. Simultaneously, the weight of the sediment, which represents the yogurt matrix, was also measured. These measurements allowed for the calculation of the WHC, which indicates the yogurt’s ability to retain water, as well as the degree of syneresis, which is the loss of whey from the yogurt matrix.

$$Water\;holding\;capacity\left(\%\right)=\frac{m_{2}}{m_{1}}\times 100$$

$$Syneresis\;(\%)=\frac{m_{3}}{m_{1}}\times 100$$

where *m*_1_ is the initial mass of the yogurt (g); *m*_2_ is the mass of the sedimentation product (g); *m*_3_ is the mass of the supernatant (g).

### 2.6. Determination of Texture, Viscosity, and Color of Yogurt

The textural attributes of the yogurt were quantified using a Texture Analyser (CT325K230, Brookfield Engineering, Middleborough, MA, USA) according to the method of Du et al. with some modifications [15]. The yogurt samples (50 mL) were used for testing at a temperature of 4 °C. The probe model is P/36R, with a diameter of 36 mm. The measurement parameters are as follows: the pre-test speed, test speed, and post-test return speed are all 1.0 mm/s; the test distance is 50%; the trigger force is 5 g; and the relaxation time is 5 s.

For the measurement of yogurt viscosity, a Rapid Visco Analyser (RVA-TecMaster, Perten, Stockholm, Sweden) was employed. The yogurt samples (50 mL) were used for testing at a temperature of 4 °C. A No. 5 rotor was used with a speed of 100 rpm and a time of 30 s.

Additionally, the color characteristics of the yogurt, namely lightness (L*), redness–greenness (a*), and yellowness–blueness (b*), were assessed using a colorimeter (ULtraScan PRO, HunterLab, Reston, VA, USA). A standard white and color board were used to standardize the colorimeter separately.

### 2.7. Determination of Antioxidant Activity of Yogurt

The yogurt was centrifuged at 4 °C and 1700× *g* for 10 min. The supernatant was filtered through a 0.45 μm filter to obtain the sample.

The determination of 2,2-diphenyl-1-picrylhydrazyl (DPPH) radical scavenging activity was performed as previously described [16]. Two hundred μL of the sample was combined with 1 mL of 100 μM DPPH and incubated in the dark at room temperature for 30 min. The absorbance was then measured at 515 nm using a spectrophotometer. Each sample was analyzed in triplicate, with distilled water serving as the blank. The DPPH radical scavenging activity (%) was calculated using the following formula:

$$DPPH\;radical\;scavenging\;activity\left(\%\right)=\frac{\left(Ac-As\right)}{Ac}\times 100$$

The 2,2′-azino-bis-3-ethylbenzotiazolin-6-sulfonic acid (ABTS) test was carried out according to earlier instructions [16]. To prepare the ABTS radical solution, mix 14.8 mM ABTS and 5 mM potassium persulfate in a 1:1 (*v*/*v*) ratio. Allow this mixture to stand at room temperature in the dark for 16 h to ensure the completion of the radical generation process. Before the assay, dilute the ABTS solution with distilled water to achieve an absorbance of approximately 0.70 ± 0.05 at 734 nm. Twenty microliter of the sample was added to 980 μL of diluted ABTS solution and incubated at 37 °C for 15 min. The absorbance was measured at 734 nm. Each sample was tested in triplicate. Distilled water was used instead of the sample as the blank. The ABTS radical scavenging activity (%) was calculated using the following formula:

$$ABTS\;radical\;scavenging\;activity(\%)=\frac{\left(Ac-As\right)}{Ac}\times 100$$

where *Ac* is the absorbance of the control (distilled water) and *As* is the absorbance of the sample.

### 2.8. Determination of Sensory Evaluation of Yogurt

The sensory attributes of the yogurt were assessed by a semi-trained panel comprising 23 individuals. The assessment utilized a nine-point hedonic scale (ranging from 9, indicating ‘like very much’, to 1, indicating ‘dislike very much’) to rate each sample based on color, flavor, texture, and odor, as well as overall acceptability. Each sample was labeled with a random three-digit code and served in a random order to each tester.

### 2.9. Statistical Analysis

The results were presented as the average of three duplicate experiments. The one-way ANOVA and Tukey’s post hoc test were applied to the statistical analysis, and significant differences were determined at *p* < 0.05.

## 3. Results and Discussion

### 3.1. Strains That Efficiently Metabolize KGM

**Figure 1:**
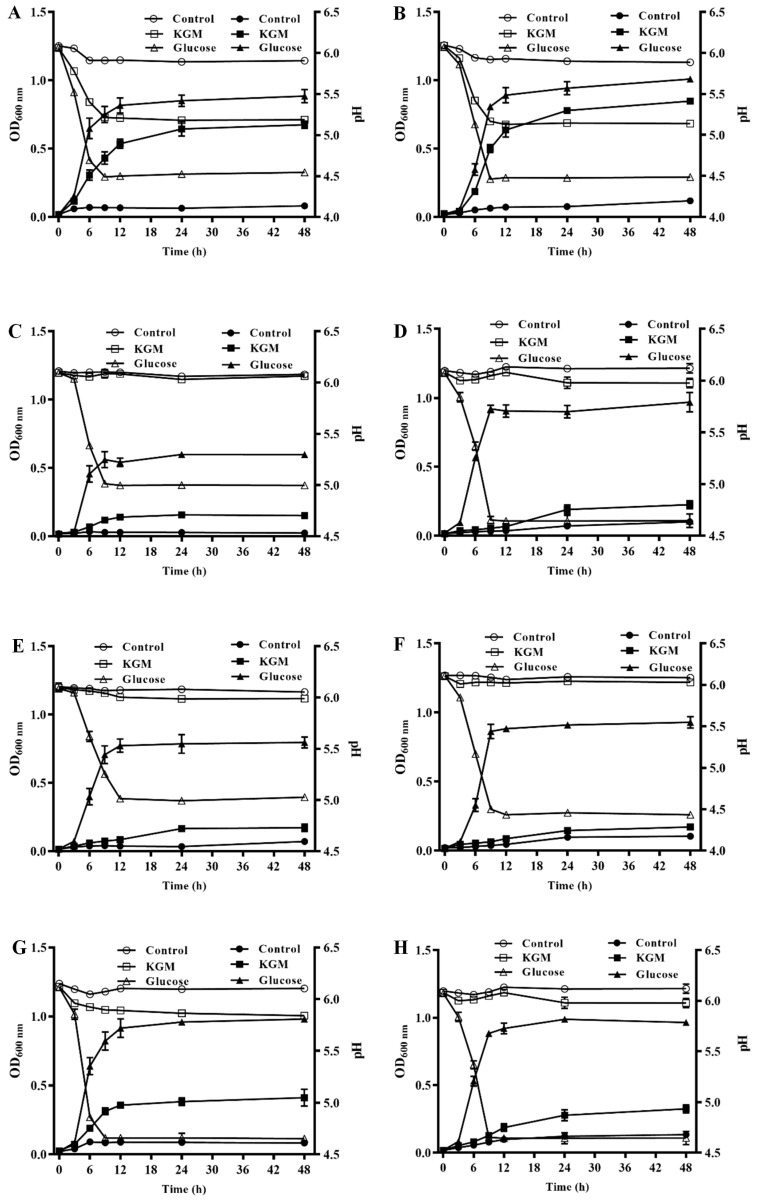
Growth curves and medium pH changes in each strain when konjac glucomannan (KGM) or glucose was the sole carbon source. (**A**) *Lactiplantibacillus plantarum* 17001, (**B**) *Lactiplantibacillus plantarum* SHY130, (**C**) *Limosilactobacillus fermentum* H131, (**D**) *Lacticaseibacillus rhamnosus* 127, (**E**) *Limosilactobacillus fermentum* 02001, (**F**) *Lacticaseibacillus casei* 01001, (**G**) *Lactiplantibacillus plantarum* S58, and (**H**) *Lacticaseibacillus rhamnosus* T53. ■, ●, and ▲ indicate the change in OD_600 nm_; □, △, and ○ indicate the change in pH.

In this study, the growth of different LAB strains under various carbon source conditions was monitored by measuring the absorbance at a wavelength of 600 nm, and the corresponding pH changes were recorded (Figure 1). In a medium devoid of a carbon source, all eight LAB strains exhibited impaired growth and an inability to produce lactic acid, leading to no significant decline in the pH of the medium. Conversely, when glucose was provided as the sole carbon source, all the strains demonstrated robust growth, reaching a stationary phase after 12 h of incubation, and concurrently produced substantial amounts of lactic acid, which rapidly decreased the pH of the culture medium. In a medium where KGM was the sole carbon source, only four strains—*Lactiplantibacillus plantarum* 17001, *Lactiplantibacillus plantarum* SHY130, *Lactiplantibacillus plantarum* S58, and *Lacticaseibacillus rhamnosus* T53—were capable of utilizing KGM and grew normally. However, the activities of these strains were not as high as those in the glucose medium, and they entered the stationary phase later, producing less lactic acid, which resulted in a small decrease in pH.

The strains that exhibited the ability to utilize KGM entered the stationary phase at a delayed stage when cultured in a KGM medium, which may be attributed to the intrinsic complexity of KGM metabolism and its relatively inefficient decomposition compared to glucose [17]. In addition, the changes in pH are consistent with the changes in OD_600 nm_, as pH changes are closely related to the growth of strains. The two strains with higher KGM utilization activity in our study are both *Lactiplantibacillus plantarum*, which is similar to the reported strong carbohydrate utilization ability of *Lactiplantibacillus plantarum* LP-F1 [18]. *Lactiplantibacillus plantarum* SHY130 is a probiotic isolated from traditional Chinese yak fermented yogurt. Our previous studies have reported that it can significantly improve body weight, hyperglycemia, glucose tolerance, and insulin resistance by regulating the enteroinsular axis [11]. Additionally, it can regulate liver metabolism and alleviate liver damage [19]. In this study, *Lactiplantibacillus plantarum* SHY130 showed stronger KGM utilization activity than the other strains and was therefore selected to be combined with KGM and added to the yogurt as a synbiotic.

### 3.2. Viable Bacterial Count, pH, and Titration Acidity of Yogurt during Storage

**Figure 2:**
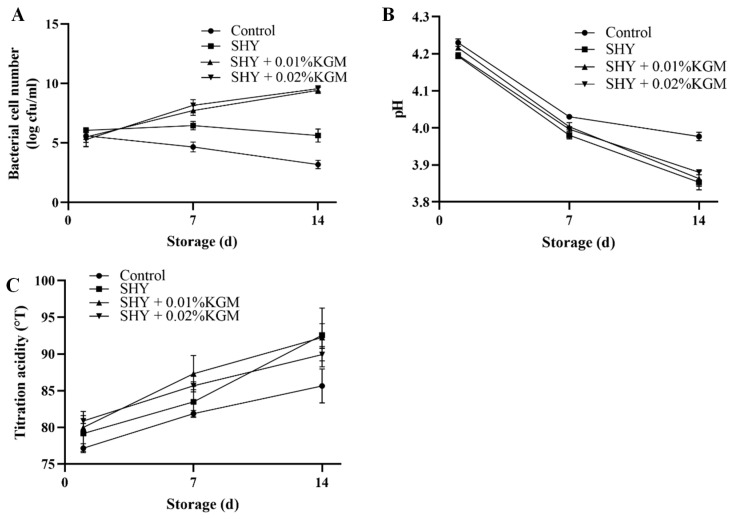
Bacterial cell numbers (**A**), pH (**B**), and titration acidity of yogurt (**C**). Control: the yogurt with only the addition of the starter culture; SHY: the yogurt with the addition of the starter culture and SHY130; SHY + 0.01% KGM: the yogurt with the addition of the starter culture, SHY130, and 0.01% KGM; SHY + 0.02% KGM: the yogurt with the addition of the starter culture, SHY130, and 0.02% KGM.

During the 14-day storage period, the dynamic changes in the number of LAB, pH value, and titration acidity in the yogurt were monitored. The results are shown in Figure 2. Throughout storage, the number of LAB in the yogurt of the control group and the SHY group gradually decreased. In contrast, the number of LAB in the yogurt with the synbiotics continued to increase over the 14-day storage period. On the 14th day, the number of viable bacteria in the synbiotic yogurt was approximately 10 log CFU/mL, while the amount of viable bacteria in the SHY group was only approximately 5 log CFU/mL. The change in the LAB numbers directly affects the pH value of the yogurt. As lactic acid continues to be generated, the pH value of the yogurt gradually decreases. Notably, the pH value of the control group decreased slowly, and on the 14th day, its pH value was significantly different from the other groups (*p* < 0.05). The change in the titration acidity of the yogurt is inversely proportional to the pH value.

The growth and reproduction of lactobacilli depend on specific nutrients, such as lactose. Therefore, the decrease in the number of LAB in the control yogurt may be due to a reduction in the supply of the nutrients necessary for their proliferation and survival. In the yogurt with synbiotics, LAB can continue to grow, which may be due to the KGM in the synbiotics serving as an energy source, thus maintaining a high level of probiotic activity [20]. Similarly, Falah et al. found that when the prebiotic inulin was added to yogurt, the viability of the probiotic *Lactobacillus brevis* PML1 increased during storage [21]. Additionally, the production of lactic acid and the primary metabolite acetic acid may cause acid damage to organisms, and the addition of prebiotics can effectively resist this acid damage, thereby ensuring the reproduction of probiotics [22]. The decrease in the pH value and increase in the titration acidity in yogurt are closely related to the growth and metabolic activities of LAB. The fluctuations observed in the pH level and titration acidity in the control group are potentially associated with the initial concentration of the probiotic cultures introduced into the milk [23].

### 3.3. The Color of Yogurt

**Figure 3:**
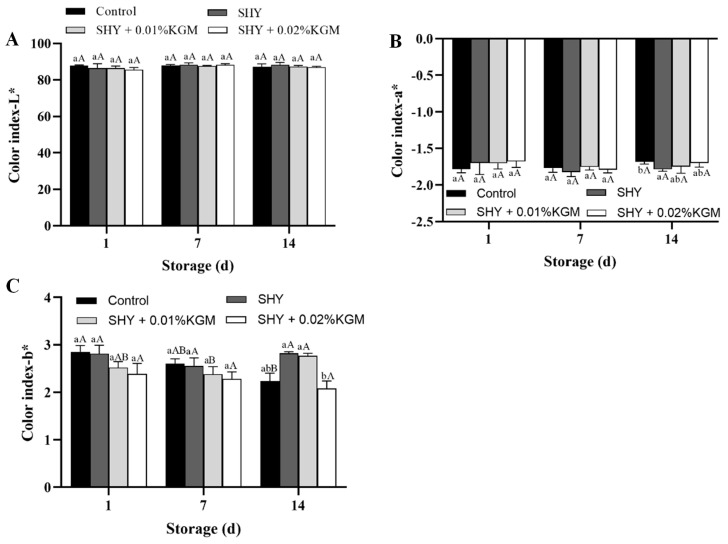
Changes in the color of yogurt during storage. (**A**) Color index L*, (**B**) color index a*, and (**C**) color index b*. Control: the yogurt with only the addition of the starter culture; SHY: the yogurt with the addition of the starter culture and SHY130; SHY + 0.01% KGM: the yogurt with the addition of the starter culture, SHY130, and 0.01% KGM; SHY + 0.02% KGM: the yogurt with the addition of the starter culture, SHY130, and 0.02% KGM. The means of varying storage times, indicated by varying uppercase letters (A,B), exhibit significant differences (*p* < 0.05); the means of varying yogurts, indicated by the lowercase letters (a,b), exhibit significant differences (*p* < 0.05).

During storage, the color properties of yogurt undergo significant changes, as shown in Figure 3. For the lightness (L*) and red–greenness (a*) values of the yogurt in the four groups, no statistically significant changes were detected (*p* < 0.05) during the 14-day period. In contrast, the yellow–blue degree (b*) of yogurt in the control group decreased, while the b* value of the yogurt in the SHY130 + 0.01% KGM group increased. The b* values of the yogurt in the SHY130 group and the SHY130 + 0.02% KGM group remained unchanged during the 14 days of storage.

The color of yogurt is an important indicator that influences its quality [24]. The alteration in the b* value during storage may be due to the utilization of riboflavin, β-carotene, and vitamin A present in milk by LAB. Changes in the number and species of LAB in different treatment groups lead to variations in the b* value of yogurt. Buchilina et al. reported that adding monk fruit sweetener to camel milk yogurt results in a decrease in b* value [25].

### 3.4. The Texture of Yogurt

The texture of the yogurt is shown in Table 1. During the initial 7 days of storage, the hardness, gumminess, and chewiness of the yogurt in all four groups increased. Subsequently, as the storage time was extended, the hardness, gumminess, and chewiness of the yogurt samples gradually decreased. Following the addition of *Lactiplantibacillus plantarum* SHY130 and KGM, the hardness, gumminess, and chewiness of the yogurt were markedly lower than those of the control group on the 1st and 14th day of storage (*p* < 0.05). However, the addition of KGM and probiotics did not result in significant alterations to the springiness and cohesiveness of the yogurt, and these textural parameters remained relatively stable throughout the storage duration.

The texture of yogurt is characterized by several key indicators: hardness, cohesiveness, gumminess, springiness, and chewiness. Hardness directly correlates with the gel state of the yogurt; higher hardness indicates better coagulation. Cohesiveness reflects the yogurt’s resistance to compression by a probe, with lower cohesiveness signifying smoother yogurt. Gumminess represents the energy needed to chew the yogurt until it is ready to be swallowed. Springiness measures how well the yogurt returns to its original height or volume after being deformed. Chewiness indicates the energy required to chew the yogurt. These parameters can be used to evaluate yogurt quality by simulating the jaw movement in the human oral cavity [26]. The early increase in hardness, gumminess, and chewiness in the synbiotic yogurt may be due to the formation of a three-dimensional network structure between KGM and protein. The latter decrease may be due to the hydrolysis and consumption of protein and KGM, making it difficult to form a larger three-dimensional network structure. This phenomenon is similar to findings from a study where increasing the supplementation of prickly pear decreased the hardness, adhesiveness, cohesiveness, and gumminess of probiotic yogurt [27]. Besides, the addition of synbiotics to yogurt appears to result in a reduction in hardness, gumminess, and chewiness, which can be attributed to KGM’s water-binding capacity and its influence on the structural integrity and stability of the food matrix.

### 3.5. WHC, Syneresis, and Viscosity of Yogurt

**Figure 4:**
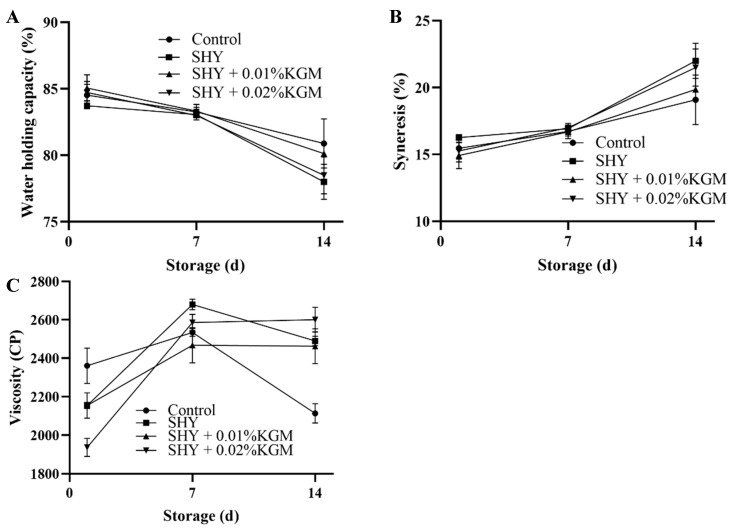
Changes in the water-holding capacity (**A**), syneresis (**B**), and viscosity (**C**) of yogurt during storage. Control: the yogurt with only the addition of the starter culture; SHY: the yogurt with the addition of the starter culture and SHY130; SHY + 0.01% KGM: the yogurt with the addition of the starter culture, SHY130, and 0.01% KGM; SHY + 0.02% KGM: the yogurt with the addition of the starter culture, SHY130, and 0.02% KGM.

The changes in the WHC, syneresis, and viscosity of each group of yogurt during storage are shown in Figure 4. The results indicate that the incorporation of probiotics and synbiotics into the yogurt did not have a significant impact on WHC and syneresis throughout the storage duration. WHC exhibited a decreasing trend as storage time progressed, whereas syneresis increased over the same period. Regarding viscosity, an initial increase was observed during the first 7 days of storage. Subsequent to this initial phase, the viscosity of yogurt in all the groups, except for the synbiotic group, began to decline.

The WHC of yogurt is an important measure of the denseness of the protein gel network and the texture of yogurt. The decrease in WHC during storage may be due to the rearrangement and shrinkage of the protein networks [28]. The increase in syneresis is related to the rearrangement of the casein network, which leads to increased interparticle binding, thus making the network prone to compression and shrinkage [29]. Furthermore, a sustained decrease in yogurt pH may also lead to the shrinkage of the casein network and higher levels of syneresis [30]. The increase in yogurt viscosity in the early stages of storage may be due to the continued formation of the protein network, while the decrease in viscosity in the later stages may be due to the destruction of the protein network by the pH value. This result is consistent with the findings of Gomes et al., who observed similar effects when adding different types of soluble fiber to high-protein yogurts [31]. The decrease in the viscosity of the synbiotic yogurts during the early stages of storage may be due to a bridging effect caused by the high concentration of KGM, which inhibits the formation of a continuous protein network, resulting in the highest viscosity in the control yogurt. Tseng et al. also found similar results, demonstrating that yogurt containing 3% fermented wine grape pomace had a lower viscosity [32]. In the middle stage, changes in viscosity may be due to the rearrangement of the protein network during storage; the effect of protein–protein interactions on viscosity exceeds that of synbiotics. As storage progresses, the consumption of KGM by microbial activity in the later stages of storage makes its contribution to the protein–polysaccharide network increasingly critical, maintaining the stability of yogurt viscosity [33].

### 3.6. Yogurt’s Antioxidant Activity

**Figure 5:**
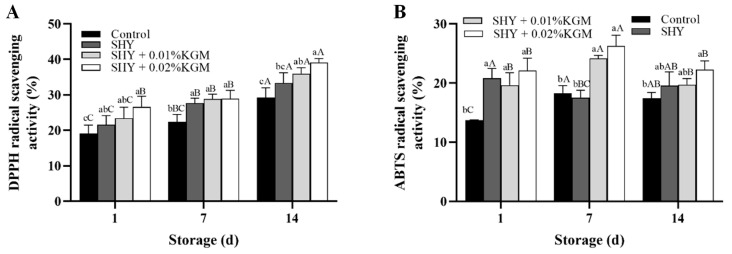
DPPH (**A**) and ABTS (**B**) free radical scavenging ability of yogurt. Control: the yogurt with only the addition of the starter culture; SHY: the yogurt with the addition of the starter culture and SHY130; SHY + 0.01% KGM: the yogurt with the addition of the starter culture, SHY130, and 0.01% KGM; SHY + 0.02% KGM: the yogurt with the addition of the starter culture, SHY130, and 0.02% KGM. The means of varying storage times, indicated by different uppercase letters (A–C), exhibit significant differences (*p* < 0.05); the means of varying yogurts, indicated by different lowercase letters (a–c), exhibit significant differences (*p* < 0.05).

During the storage period, the changes in the DPPH free radical scavenging ability and ABTS free radical scavenging ability of each group of yogurt are shown in Figure 5. The results indicate that the DPPH free radical scavenging activity in the yogurt samples progressively increased with the duration of storage. Specifically, the yogurt samples supplemented with synbiotics exhibited higher scavenging activity compared to the control group. The DPPH scavenging activity in the SHY130 + 0.02% KGM group reached the highest level on the 14th day (39.08 ± 1.17%). In contrast, the ABTS scavenging activity in the SHY130 + 0.02% KGM group peaked on the 7th day (25.21 ± 0.24%).

Compared with the yogurt without *Lactiplantibacillus plantarum* SHY130, the yogurt containing *Lactiplantibacillus plantarum* SHY130 demonstrated more efficient scavenging activity in both the DPPH and ABTS free radical scavenging experiments. This phenomenon may be attributed to the ability of *Lactiplantibacillus plantarum* SHY130 to produce a variety of antioxidant enzymes (such as superoxide dismutase, catalase, glutathione reductase, and thioredoxin) that scavenge free radicals, thereby improving the overall antioxidant capacity of yogurt [34]. Additionally, the antioxidant activity of yogurt may originate from bioactive peptides produced during the fermentation process, with milk protein being an important source of these bioactive peptides. Sah et al. purified antioxidant peptides from probiotic yogurt supplemented with pineapple peel powder and identified two peptides derived from β-casein, namely 193YQEPVLGPVRGPFPPIV209 and 69SLPQNIPPLTQTPVVVPPF87 [35]. The addition of different concentrations of KGM enhanced the antioxidant capacity of yogurt, possibly because KGM can increase the activity and quantity of *Lactiplantibacillus plantarum* SHY130 in yogurt. Our results are consistent with the research results of Sha et al., who found that supplementing yogurt with pineapple peel or inulin increased its antioxidant activity [36]. The different antioxidant activities of synbiotic yogurts suggest that free radical scavenging activity may depend on strain characteristics and that there may be differences in reaction kinetics between different free radicals and antioxidants [37].

### 3.7. Sensory Properties of Yogurt

The sensory evaluation outcomes for the yogurt samples are presented in Table 2. The statistical tests revealed that the incorporation of the synbiotics did not result in statistically significant alterations in the sensory attributes of the yogurt, including the color, flavor, texture, and odor (*p* > 0.05). Additionally, the sensory evaluation panel identified a significant decline in the overall acceptance of the yogurt samples enriched with 0.02% KGM (*p* < 0.05). These data suggest that the synbiotic yogurts with 0.01% KGM may represent an optimal compromise, preserving the sensory appeal and quality attributes of the product.

## 4. Conclusions

In this study, we screened *Lactiplantibacillus plantarum* SHY130 for its high bioavailability activity towards KGM. This strain was combined with KGM and added to yogurt, and their effects on the quality and sensory properties of yogurt were evaluated. The addition of KGM increased the activity of probiotic bacteria, as evidenced by the viable bacterial count and pH of the yogurt. The synbiotics reduced the hardness, gumminess, and chewiness of the yogurt texture. Additionally, the synbiotic supplementation did not significantly impact the water-holding capacity and syneresis of the yogurt. However, it may have an adverse effect on the viscosity of yogurt in the early stages of storage, but it increases the viscosity in the later stages. Furthermore, the synbiotics significantly improved the antioxidant properties of the yogurt. Although the addition of the synbiotics did not affect the sensory properties, the overall acceptability of the synbiotic yogurt containing 0.02% KGM was low. Taken together, these results provide a new reference and approach for the development of new synbiotic yogurt.

## Figures and Tables

**Table 1:** Changes in the texture parameters of the yogurt during storage.

Parameters	Treatment	Storage Period (Days)		
		1	7	14
Hardness (g)	Control	57.90 ± 2.55 ^aB^	75.05 ± 2.01 ^aA^	67.23 ± 1.02 ^aAB^
	SHY	53.68 ± 1.62 ^aB^	75.38 ± 0.92 ^aA^	64.15 ± 4.14 ^abB^
	SHY + 0.01%KGM	43.43 ± 2.42 ^bB^	73.35 ± 2.08 ^aA^	61.92 ± 3.69 ^abB^
	SHY + 0.02%KGM	43.75 ± 0.25 ^bC^	62.19 ± 0.46 ^bA^	57.29 ± 1.16 ^bB^
Springiness	Control	0.93 ± 0.00 ^aA^	0.94 ± 0.00 ^aA^	0.94 ± 0.00 ^aA^
	SHY	0.93 ± 0.00 ^aA^	0.93 ± 0.00 ^aA^	0.92 ± 0.02 ^aA^
	SHY + 0.01%KGM	0.93 ± 0.00 ^aA^	0.93 ± 0.00 ^aA^	0.93 ± 0.01 ^aA^
	SHY + 0.02%KGM	0.93 ± 0.00 ^aA^	0.93 ± 0.00 ^aA^	0.95 ± 0.00 ^aA^
Cohesiveness	Control	0.67 ± 0.01 ^aA^	0.60 ± 0.00 ^aA^	0.60 ± 0.01 ^aA^
	SHY	0.67 ± 0.01 ^aA^	0.61 ± 0.00 ^aA^	0.60 ± 0.02 ^aA^
	SHY + 0.01%KGM	0.68 ± 0.02 ^aA^	0.63 ± 0.01 ^aA^	0.60 ± 0.02 ^aA^
	SHY + 0.02%KGM	0.69 ± 0.00 ^aA^	0.63 ± 0.01 ^aA^	0.60 ± 0.00 ^aA^
Gumminess	Control	39.60 ± 1.28 ^aB^	45.18 ± 1.05 ^aA^	40.95 ± 0.35 ^aB^
	SHY	36.10 ± 1.37 ^aB^	46.08 ± 1.20 ^aA^	37.62 ± 1.03 ^abB^
	SHY + 0.02%KGM	29.70 ± 1.07 ^bC^	45.59 ± 0.91 ^aA^	35.43 ± 0.76 ^bB^
	SHY + 0.02%KGM	30.23 ± 0.17 ^bC^	40.50 ± 0.40 ^bA^	35.63 ± 0.48 ^bB^
Chewiness	Control	37.16 ± 1.19 ^aB^	42.45 ± 0.98 ^aA^	38.64 ± 0.56 ^aB^
	SHY	33.81 ± 1.31 ^aB^	43.13 ± 1.16 ^aA^	34.80 ± 0.60 ^abB^
	SHY + 0.01%KGM	27.82 ± 1.02 ^bC^	42.57 ± 0.91 ^aA^	33.01 ± 0.49 ^bB^
	SHY + 0.02%KGM	28.24 ± 0.16 ^bC^	38.15 ± 0.03 ^bA^	33.67 ± 0.37 ^bB^

Control: the yogurt with only the addition of the starter culture; SHY: the yogurt with the addition of the starter culture and SHY130; SHY + 0.01% KGM: the yogurt with the addition of the starter culture, SHY130, and 0.01% KGM; SHY + 0.02% KGM: the yogurt with the addition of the starter culture, SHY130, and 0.02% KGM. The means of varying storage times, indicated by different uppercase letters (A–C), exhibit significant differences (*p* < 0.05); the means of varying yogurts, indicated by different lowercase letters (a,b), exhibit significant differences (*p* < 0.05).

**Table 2:** The sensory scores of yogurt after 7 days.

Sample	Color	Flavor	Texture	Odor	Overall Acceptability
Control	7.57 ± 1.08 ^a^	7.15 ± 1.47 ^a^	7.21 ± 1.04 ^a^	7.15 ± 1.29 ^a^	7.46 ± 0.81 ^a^
SHY	7.54 ± 1.05 ^a^	6.85 ± 1.61 ^a^	7.33 ± 0.93 ^a^	7.33 ± 1.44 ^a^	7.17 ± 1.30 ^ab^
SHY + 0.01%KGM	7.17 ± 1.30 ^a^	6.78 ± 1.48 ^a^	7.41 ± 1.21 ^a^	6.85 ± 1.29 ^a^	7.20 ± 1.21 ^ab^
SHY + 0.02%KGM	7.20 ± 1.44 ^a^	6.35 ± 1.78 ^a^	7.13 ± 1.10 ^a^	6.74 ± 1.71 ^a^	6.67 ± 1.38 ^b^

Note, control: the yogurt with only the addition of the starter culture; SHY: the yogurt with the addition of the starter culture and SHY130; SHY + 0.01% KGM: the yogurt with the addition of the starter culture, SHY130, and 0.01% KGM; SHY + 0.02% KGM: the yogurt with the addition of the starter culture, SHY130, and 0.02% KGM. Means within a column with different superscripts (a,b) differ significantly (*p* < 0.05).

## Data Availability

The original contributions presented in the study are included in the article, further inquiries can be directed to the corresponding author.
